# Whole Genome Analysis of a Wine Yeast Strain

**DOI:** 10.1002/cfg.73

**Published:** 2001-04

**Authors:** Nicole C. Hauser, Kurt Fellenberg, Rosario Gil, Sonja Bastuck, Jörg D. Hoheisel, José E. Pérez-Ortín

**Affiliations:** 1 Functional Genome Analysis Deutsches Krebsforschungszentrum Im Neuenheimer Feld 506 Heidelberg D-69120 Germany; 2 Theoretical Bioinformatics Deutsches Krebsforschungszentrum Im Neuenheimer Feld 506 Heidelberg D-69120 Germany; 3 Departamento de Bioquímica y Biología Molecular and Servicio de Chips de DNA Universitat de València Dr. Moliner 50 Burjassot E-46100 Spain

## Abstract

*Saccharomyces cerevisiae* strains frequently exhibit rather specific phenotypic features needed for adaptation to a special environment. Wine yeast strains are able to ferment musts, for example, while other industrial or laboratory strains fail to do so. The genetic differences that characterize wine yeast strains are poorly understood, however. As a first search of genetic differences between wine and laboratory strains, we performed DNA-array analyses on the typical wine yeast strain T73 and the standard laboratory background in S288c. Our analysis shows that even under normal conditions, logarithmic growth in YPD medium, the two strains have expression patterns that differ significantly in more than 40 genes. Subsequent studies indicated that these differences correlate with small changes in promoter regions or variations in gene copy number. Blotting copy numbers vs. transcript levels produced patterns, which were specific for the individual strains and could be used for a characterization of unknown samples.


**Comparative and Functional Genomics**
*Comp Funct Genom* 2001; **2**: 69–79.
**DOI**: 10.1002/cfg.73

**Research Article**

### Whole genome analysis of a wine yeast strain

Nicole C. Hauser[1], Kurt Fellenberg[1,2], Rosario Gil[3], Sonja Bastuck[1], Jörg D. Hoheisel[1] and José E. Pérez-Ortín[1,3]*

[1] *Functional Genome Analysis, Deutsches Krebsforschungszentrum, Im Neuenheimer Feld 506, D-69120 Heidelberg, Germany*
[2] *Theoretical Bioinformatics, Deutsches Krebsforschungszentrum, Im Neuenheimer Feld 506, D-69120 Heidelberg, Germany*
[3] *Departamento de Bioquímica y Biología Molecular and Servicio de Chips de DNA, Universitat de València, Dr. Moliner 50, E-46100, Burjassot, Spain*

**Correspondence to: J. E. Pérez-Ortín, Departamento de Bioquímica y Biología Molecular, Universitat de Valencia, Dr. Moliner 50, E-46100, Burjassot, Spain. E-mail: jose.e.perez@uv.es*

Received: 20 November 2000
Accepted: 21 February 2001

### Abstract

***Saccharomyces cerevisiae* strains frequently exhibit rather specific phenotypic features needed for adaptation to a special environment. Wine yeast strains are able to ferment musts, for example, while other industrial or laboratory strains fail to do so. The genetic differences that characterize wine yeast strains are poorly understood, however. As a first search of genetic differences between wine and laboratory strains, we performed DNA-array analyses on the typical wine yeast strain T73 and the standard laboratory background in S288c. Our analysis shows that even under normal conditions, logarithmic growth in YPD medium, the two strains have expression patterns that differ significantly in more than 40 genes. Subsequent studies indicated that these differences correlate with small changes in promoter regions or variations in gene copy number. Blotting copy numbers vs. transcript levels produced patterns, which were specific for the individual strains and could be used for a characterization of unknown samples.** **Copyright © 2001 John Wiley & Sons, Ltd.**

**Keywords:** ***Saccharomyces cerevisiae*; wine yeast; genome; transcriptome; DNA arrays**

### Introduction

*Saccharomyces cerevisiae* wine yeast strains have been selected for more than 8000 years by human beings under conditions that favoured the evolution towards several specialized features, such as fast growth in high-sugar fruit juices, high yield of and resistance to ethanol and, more recently, sulphite resistance and good flavour production (reviewed in Querol and Ramón, 1996; Pretorius, 2000). Wine yeasts exhibit a greater variety in chromosome size and number than reported for laboratory strains. They are aneuploid (Kunkee and Bisson, 1993; Bakalinsky and Snow, 1990), with disomies or trisomies and, in some cases, they are near diploid or triploid. The aneuploidy can confer selective advantages by increasing the copy number of beneficial genes and protecting against lethal or deleterious mutations (Guijo *et al.*, 1997; Bakalinsky and Snow, 1990). Aneuploidy and/or polyploidy are so widespread that the maintenance of an unbalanced chromosome set has been suggested to be advantageous (Sancho *et al.*, 1986; Adams *et al.*, 1992; Codón *et al.*, 1998). Chromosomal changes include gain or loss of chromosomes, deletions (30–50 kb), presence of hybrid chromosomes (Bidenne *et al.*, 1992; Rachidi *et al.*, 1999) and, in most cases, duplications (30–390 kb) (Adams *et al.*, 1992). It has been suggested that these rearrangements occurred by recombination through subtelomeric repeats and transposable elements (Ty elements) (Rachidi *et al.*, 1999, and references therein). Minor differences, such as point mutations, are also possible. Such variations may also affect strain performance, if they localize inside an open reading frame (ORF) or regulatory regions of a gene, especially if it is a pathway regulating one (Cavalieri *et al.*, 2000).

There are very few DNA sequences of wine yeasts published or stored in databases (Goto-Yamamoto *et al.*, 1998; Masneuf *et al*, 1998) but it seems that,

Copyright © 2001 John Wiley & Sons, Ltd.

overall, the sequence homology between the standard laboratory yeast strain S288c (whose genome was fully sequenced by Goffeau *et al.*, 1996) and wine yeasts is higher than 99% (Masneuf *et al.*, 1998; Pérez-Ortín, unpublished observations). This fact should permit the systematic use in wine yeast of probes and primers obtained or designed from the S288c sequence (Puig *et al.*, 1998, 2000a). DNA arrays made of S288c sequences have been used in recent years for the analysis of many cellular processes in yeast (DeRisi *et al.*, 1997; Wodicka *et al.*, 1997; Holstege *et al.*, 1998; Jelinski and Samson, 1999), but usually with S288c or genetically related strains. However, when using long PCR products as probes for hybridization (DeRisi *et al.*, 1997; Hauser *et al.*, 1998; Cox *et al.*, 1999), point mutations cannot be discriminated. Therefore, this kind of DNA array should also work in the analysis of non-laboratory yeast strains (Cavalieri *et al.*, 2000).

In this study, the comprehensive gene arrays of Hauser *et al.* (1998) were used for the analysis of both the genomic copy number and transcript levels of wine yeast T73. Even under standard laboratory growth conditions, the transcription profiles of wine and laboratory strains differed significantly for several genes, some of which can be related to special features of wine yeast physiology. Three such genes, *YHB1*, *SSU1* and *YJL217w*, have been analysed in greater detail. Also, both transcript and genome analyses show that wine strain T73 is much less colonized by Ty transposons than S288c.

### Materials and methods

### Strains and growth conditions

The *S. cerevisiae* yeast strains used in this study are FY1679 (*MAT***a**/α, *ura3-52/ura3-52*, *trp1-Δ1/TRP1*, *leu2-3/LEU2*, *his3-Δ200/HIS3*) and MCY730 (*MAT***a**, *ura3-52*, *lys2-801*, *ade2-101*, *trp1-Δ1*, *his3-Δ200*) as laboratory strains, as well as T73, a wine strain commercialized by Lallemand Inc. (Montreal, Canada) (Querol *et al.*, 1992). Yeast cells were grown at 30°C in YPD rich medium (1% Yeast Extract, 2% Bacto-peptone, 2% glucose). Solid medium was supplemented with 2% agar.

### Nomenclature note

We call 'probes' the pieces of DNA tethered to the array and 'target' the free, labelled DNA-fragments hybridized to the probes. Each probe-DNA was spotted in duplicate; the two spots are referred to as 'primary' and 'secondary' spot to indicate that they represent the same ORF.

### Nucleic acids isolation and hybridization

Genomic DNA was isolated from yeast cells by phenol extraction with glass beads, essentially as described by Hoffman and Winston (1987) or with QIAGEN genomic DNA spin columns, as recommended by the manufacturer.

Total RNA from yeast cells was prepared as described by Sherman *et al.* (1986), but using a multiple-sample automated device (Fast-Prep, BIO101, Inc.) to break the cells, or following the procedure of Hauser *et al.* (1998).

All arrays were subjected to a standard control procedure prior to being used in experimentation. First, an oligonucleotide was hybridized that specifically bound to the forward primers of all PCR products. After removal of the oligonucleotide, total genomic DNA from a specific preparation was applied as a second control. While the former hybridization determined accurately the amount of DNA present at each spot, the latter experiment served as an indicator for the usefulness of the individual arrays in complex hybridizations.

Labelling of total RNA by reverse transcription was done as described (Hauser *et al.*, 1998). Genomic DNA was sonicated to fragments and labelled by random priming. Hybridization with either target was performed according to the protocol used for labelled cDNA (Hauser *et al.*, 1998).

### Signal acquisition and analysis

Digital images of radioactive signals were acquired with Molecular Dynamics Storm 860 or Fuji FLA3000 phosphorimagers and quantified using the ArrayVision module of AIS (Imaging Research Inc.). Signal intensities of repeated hybridizations were normalized and significance levels assessed by two stringency criteria, as described by Beissbarth *et al.* (2000). The highly stringent 'min–max separation' is calculated by taking the minimum distance between all data points of the two strains. The less stringent criteria, called 'standard deviation separation', is defined as the difference of the means of the two data sets diminished by one standard deviation. In the tables, a colour code indicates the two stringency measures.

Copyright © 2001 John Wiley & Sons, Ltd. 

### Northern blotting

Northern blot analysis was made on RNA samples that were separated by electrophoresis in formaldehyde-containing agarose gels (Sambrook *et al.*, 1989). The DNA-transfer to nylon membranes (Hybond-N+, Amersham) was carried out according to the manufacturer's instructions.

PCR amplification of DNA from strain T73 was made as follows: the PCR mixture was prepared with 200 ng genomic DNA as template, appropriate primer molecules (60 pmol each), 0.2 mM each dNTP, BioTaq buffer, 2.5 mM $MgCl_2$ and 2 U BioTaq (BioLine, UK) in a 50 μL volume. The reaction conditions were: 25 cycles of 30 s at 94°C, 30 s at 50°C and 30 s at 72°C, followed by a final incubation of 3 min at 72°C. PCR products were isolated after agarose gel electrophoresis by the Gene Clean (Bio101 Inc.) procedure and used for probe labelling by random priming or DNA sequencing.

### Results

### Use of S288c arrays for wine yeast analysis

Several highly stringent hybridizations on different arrays were performed consecutively with random-primed genomic DNA from laboratory and wine strains. The total number of detectable spots was identical within experimental variation, documenting that no major differences exist between the T73 wine yeast and the S288c genetic background. We concluded that overall the existing arrays are well suited for the analysis of yeast strains other than S288c. However, some reproducible differences in the intensity of several individual spots could be seen, as will be discussed below.

### Comparative transcriptional analysis of yeast strains MCY730 and T73

Total RNA was obtained from either strain T73 or strain MCY730 (which has a S288c genetic background) grown with orbital agitation in YPD medium to middle logarithmic phase ($OD_{600} = 0.5-0.6$). Repeated hybridizations were performed with material of both strains, resulting in six data sets each, considering primary and secondary spots individually. Relative changes between T73 and MCY730 and a measure of the significance level were calculated, the latter indicated by a colour code, as shown in Table 1. The complete list for all ORFs as well as the median of normalized signal intensities can be downloaded from our web pages: **http://scsie.uv.es/chipsdna/** and **http://www.dkfz-heidelberg.de/funct_genome/index.html**. An overview of the transcript levels is presented in Figure 1. A list of selected genes, which either exhibited the most significant changes or proved to be of particular interest, is given in Table 1.

All of the Ty1 and Ty2 elements present on the array showed higher transcript levels in the laboratory strain MCY730 compared to T73 (Table 2). Every Ty encodes for two genes called TyA and TyB (Boeke and Sandmeyer, 1991). Since the Ty1 transposons are very similar in DNA sequence, the signal at each spot could be considered as an average of the contribution of all these transcripts. We observed much higher differences for Ty1B ORFs compared to Ty1A ORFs, the average ratio MCY730 : T73 being 38.8 and 8.6, respectively. Moreover, not only relative transcriptional changes are higher for Ty1B, but also the normalized signal intensities (see Table 2). The other Ty elements, except for one case of Ty2B, gave rise to signal intensities below the significance threshold and thus were not considered.

Another gene that appears to be higher expressed in MCY730 is *URA3*. Since the strain is *ura*$^-$, it is not able to synthesize uracil. However, the mutation *ura3-52* is a transposon insertion into the ORF of the wild-type gene (Brachmann *et al.*, 1998) and does not block transcription. This explains why a signal was detected. *URA1*, which functions downstream in the biosynthesis of uracil, also produced higher transcript levels in the *ura3*$^-$ laboratory strain. Its transcription could be affected by a regulation deficiency in the biochemical pathway because of the lack of one enzyme. Similar explanations could be true for the genes involved in lysine and adenine biosynthesis, because the MCY730 strain is *lys2*$^-$ and *ade2*$^-$. Similar results have been described for the BY4743 strain, which is *his3*$^-$ and *leu2*$^-$ (Hughes *et al.*, 2000).

Two genes coding for **a**-factor specific proteins, *MFa1* and *MFa2*, also exhibited high expression in MCY730. They are **a**-specific and should therefore be expressed in *MAT***a** strains, such as MCY730, and not expressed in diploid strains, such as T73. It is interesting to note that, in spite of comparing a haploid strain (MCY730) to a near-diploid one (T73), we did not find significant differences

Copyright © 2001 John Wiley & Sons, Ltd. 

between the genes described by Galitski *et al.* (1999) as differentially expressed in dependence on the level of ploidy.

Another 23 of the genes shown in Table 1 are known to be involved in a variety of functions such as amino acid biosynthesis, purine biosynthesis and stress responses. Two of them seemed to be particularly interesting, *SSU1* and *YHB1*. *SSU1* encodes for a plasma membrane protein involved in sulphite resistance (Park and Bakalinsky, 2000). This gene had been previously shown to be differentially expressed in wine yeast strains other

Copyright © 2001 John Wiley & Sons, Ltd. 

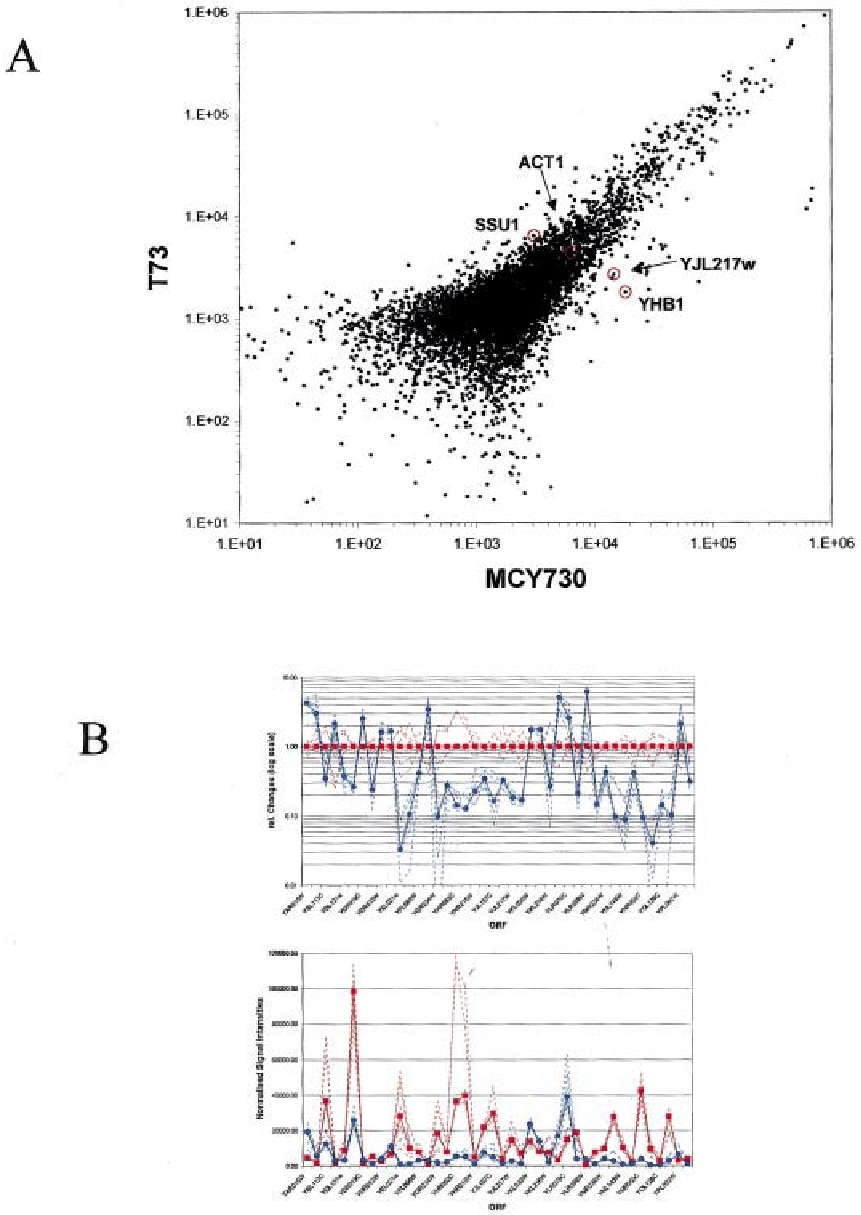

**Figure 1.** Comparison of transcript levels in strains T73 and MCY730. (A) Scatterplot of the median of the normalized signal intensities. Each black dot represents a gene. Indicated genes are presented as Northern blot analyses in Figure 2. (B) Presentation of the relative changes in transcript signals (top) and the normalized signal intensities (bottom) of particular ORFs. Dotted lines represent individual measurements, **bold** lines the respective mean value than T73 (Goto-Yamamoto *et al.*, 1998). *YHB1* encodes a flavohaemoglobin, whose expression is related to the presence of $O_2$ (Liu *et al.*, 2000). Both genes were analysed in greater detail (see below).

The remaining 10 differentially transcribed genes fall into the category of orphans. Two of them, *YPR203w* and *YBL113c*, belong to families of subtelomeric proteins, many members of which show partial homologies. Their signal intensities could, at least in part, be the result of cross-hybridization events between the transcripts of the members of their families. Another orphan gene, *YJL217w*, is analysed in greater detail below.

Copyright © 2001 John Wiley & Sons, Ltd. 

### Northern hybridization analysis of selected genes

In order to confirm the results obtained with the arrays, we performed Northern hybridization analysis with probes specific for some of the selected genes. Figure 2 shows typical results for three genes: *SSU1*, *YHB1* and *YJL217w*, with *ACT1* acting as control. In all three cases, differences were observed, which are in agreement with the array results: *SSU1* is higher expressed in T73; *YHB1* and *YJL217w* were clearly expressed in MCY730 while their transcript levels were not detectable in T73.

### Sequence analysis of some wine yeast promoters

There are several possible explanations for a gene to be differentially expressed in two strains. An obvious one is the presence of differences in the promoter sequence. To test this possibility, we chose few of the genes reported above. Oligonucleotide primers were designed to amplify by PCR the promoter regions from T73. The resulting fragments were then sequenced. In the case of *YHB1*, the sequence of the T73 promoter was very close to that of S288c, with only five differences in the amplified 577 bp region. All those differences were point mutations or single-base deletions (see Figure 3) except for one, an 8 bp deletion inside a 22 bp AT repeat placed 100 bp upstream of the ATG codon. Although there is no indication of a TATA box or other regulatory elements, it is tempting to speculate that the shortening of the AT repeat could explain the lower level of expression of *YHB1* in T73. We also sequenced the *SSU1* promoter. It exhibited a gross rearrangement (J. E. Pérez-Ortín and S. Puig, in preparation) identical to that observed for another wine strain, which was shown to increase sulphite resistance four-fold (Goto-Yamamoto *et al.*, 1998).

Copyright © 2001 John Wiley & Sons, Ltd.

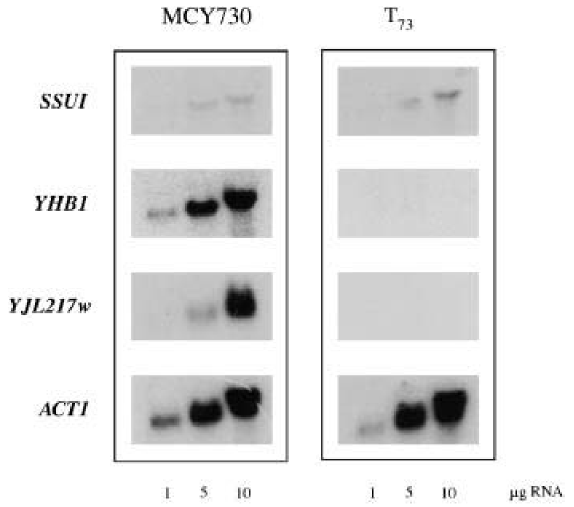

**Figure 2.** Northern hybridization analysis of the laboratory yeast strain MCY730 and the wine yeast strain T73 with probes of the genes *SSU1*, *YHB1*, *YJL217w* (differentially expressed) and *ACT1* (loading control). Increasing amounts of total RNA (1, 5 and 10 µg) per lane were used in each analysis. Expression results obtained with DNA arrays could be reproduced and confirmed by this method

### Strain characterization by comparisons of genome vs. transcriptome

We plotted the values obtained with RNA targets (transcriptome) against genomic signal intensities (genome) (Figure 4) using data from successive hybridizations, using the very same filter array in order to avoid experimental bias as much as possible. In a scatterplot, such analysis produces a croissant-shaped cloud of dots that seems to be characteristic for each strain. Dots on the top-left corner of the cloud correspond to genes that are highly expressed but have low copy numbers (1–2 copies per haploid genome), such as ribosomal proteins or glycolytic enzymes. Dots on the bottom-right corner of the cloud correspond to genes with high values in genomic hybridization and low levels of expression; in Figure 4, we highlighted in this area a gene family composed of about 30 members with more than 90% similarity in protein sequence; *YPR203w* belongs to this family. Dots located at the top-right corner correspond to genes with both high copy number and high expression levels. Between the two strains analysed here, a clear pattern difference can be seen for the Ty1 family, for example most dots are clustered in top-right corner for FY1679 as opposed to those of T73, corroborating the results obtained for the MCY730/T73 comparison (Table 2).

### Discussion

The initial goal of this study was to determine whether the yeast arrays generated with S288c DNA sequences (Hauser *et al.*, 1998) were useful for the analysis of industrial yeast strains. For this purpose, the natural isolate T73 wine yeast strain (Querol *et al.*, 1992) was used, with genomic DNA of the laboratory strains FY1679 and MCY730 acting as controls. In all cases, not all the probes produced a signal. However, the number of those that could be detected was very similar. From this, we conclude that most of the S288c genes are present in the wine strain T73. A similar conclusion can be obtained from a recent experiment on glass microarrays (Cavalieri *et al.*, 2000). As a matter of course, it is possible that this particular wine strain or other industrial yeast strains contain genes which are not present at all or in a different copy number in the standard laboratory strains. This is known, for example, for most members of the families for *SUC* (Carlson and Botstein, 1983; Naumov *et al.*, 1996), *MEL* (Naumov *et al.*, 1990) and *MAL* (Naumov *et al.*, 1994) and a gene that encodes for resistance to toxicity in molasses (Ness and Aigle, 1995). Most of these are translocated repeats of the original locus. However, they should represent only a very small fraction of the entire yeast genome. In our hands, a genomic hybridization of random-primed total DNA-samples proved to be a good procedure to detect differences in the quality of DNA arrays. This way of array validation is more sensitive than the hybridization of oligonucleotide tags, which bind to all the PCR products (Hauser *et al.*, 1998).

The comprehensive gene arrays were used for the analysis of both the genomic copy number and transcript levels of wine yeast T73. RNA was obtained from yeast cells growing in middle logarithmic phase. This comparison has some possible drawbacks. The first is the growth rate. As usual for most industrial strains, T73 grows twice as fast as MCY730 (J. Gimeno, personal communication). This means that the selected conditions might not really be identical for both strains. However, this discrepancy is unavoidable when comparing strains that are so different. One

Copyright © 2001 John Wiley & Sons, Ltd.

**Figure 3.** Comparison of the promoter sequence of gene *YHB1* in T73 and the S228c sequence stored in the MIPS database (**http://www.mips.biochem.mpg.de/**). Sequence differences and the ATG codon are marked. The GenBank Accession No. for the *YHB1* T73 sequence is AF239759

way to overcome this problem would be to perform the experiments using chemostat cultures, in which the growth rate of each strain could be controlled.

Another problem is the fact that MCY730 is a haploid strain while T73 is near-diploid (Puig *et al.*, 1998, 2000b). This has been described to affect the expression of about a dozen genes (Galitski *et al.*, 1999). We could not detect significant differences in this set of genes in our experiments. However, in our study both strains are of very different genetic background, rather than one being a duplication of the other, as was the case in the published study. This might have obscured ploidy differences.

Finally, some genes are known to be defective in MCY730. For instance, S288c background strains lack the transcription factor Flo8p (Liu *et al.*, 1996). Whether or not this gene is active in T73 is unknown, but the flocculation properties of wine yeasts are very variable (discussed in Pretorius, 2000), so it is difficult to anticipate the differences

Copyright © 2001 John Wiley & Sons, Ltd.

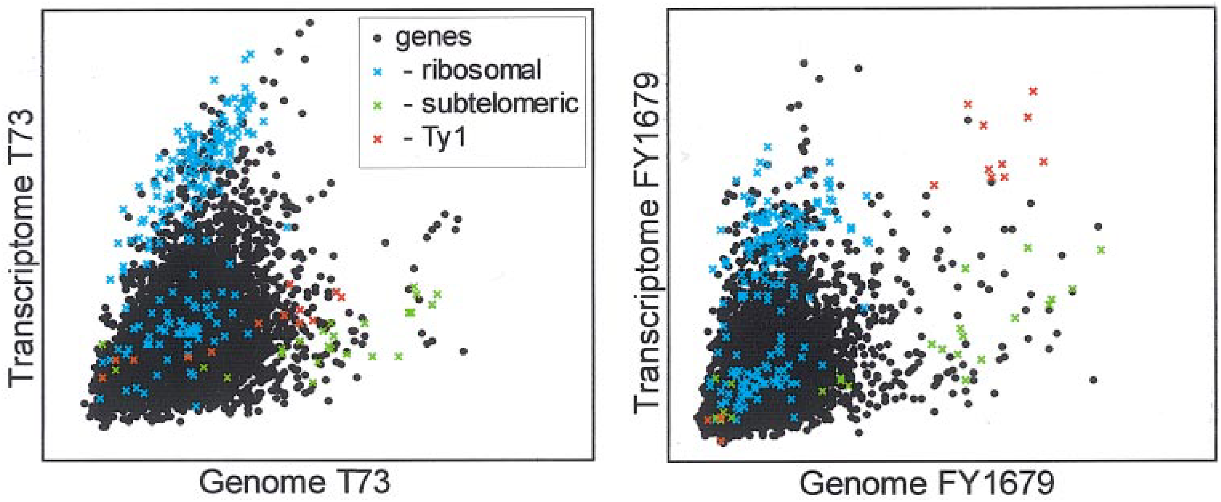

**Figure 4.** Strain characterization by blotting genomic copy numbers vs. transcript levels. Signal intensities obtained from random primed genomic DNA are plotted vs. results of hybridizations with cDNA representing total RNA preparations. Each black dot represents one gene. Gene groups of particular interest are colour-coded as indicated between MCY730 and T73. Our analysis does not show any significant variation in either *FLO8* or its targets, except for the case of *FLO1*, which is transcribed very poorly in T73 (32 times less than in MCY730), although the statistical confidence for this result only meets the less stringent quality criteria (see web pages). Other genes that differ between both strains are the ones related with *MATα* mating type or with the MCY730 auxotrophies, such as *ura3*$^{-}$. These differences (see Results) serve as an internal control of data quality. For some of the selected genes, the results from the array analysis were confirmed by Northern blot experiments. In such cases, independently isolated RNA preparations were used in order to perform biologically meaningful control experiments. As described by others (Ter Linde *et al.*, 1999), the overall expression differences found in array hybridizations were lower than those on Northern blots. In two cases (*YHB1*, *YJL217w*), we could not detect any signal on the T73 RNA samples.

We chose some particular genes for further analysis because of their special interest to wine yeasts. *SSU1* has been demonstrated to code for a plasma membrane protein required for sulphite efflux (Park and Bakalinsky, 2000). Sulphite is a widely used preservative in wine production (Pretorius, 2000). The expression level of this gene directly correlates with sulphite resistance of the yeast strain (Goto-Yamamoto *et al.*, 1998; Park and Bakalinsky, 2000). *YHB1* codes for a flavohaemoglobin whose function is not well understood. It seems to protect cells against the damage caused by nitrosylation (Liu *et al.*, 2000). Its expression is elevated in aerobic conditions (Liu *et al.*, 2000; Zhao *et al.*, 1996). Thus, this gene seems to be more important for cells growing in $O_2$-rich media. Its low expression in wine yeast may be relevant to physiological features of a strain, which has evolved for millions of generations under the $O_2$-limiting conditions of wine fermentation.

The main difference between the strains in Ty1 and Ty2 transposable elements seems to be the fact that the S288c laboratory genetic background has many more copies of Ty1 transposons than the T73 wine strain. This result confirms previous reports that Ty1 and Ty2 transposable elements are less frequent in wine and brewer's yeasts than in laboratory and, especially, bakers' strains (Codón *et al.*, 1998). The observation is also compatible with the suggestion that in most or even all the Ty elements transposed recently, and in some situations perhaps in wine fermentations, a selective pressure against accumulation of Ty elements might exist (Jordan and McDonald, 1999). Since the differences at transcript level are more pronounced than at genomic level, it is possible that Ty transcription levels are either higher in each copy of Ty1 or that some Ty1 copies are silent in the wine strain. Ploidy may also significantly alter Ty

Copyright © 2001 John Wiley & Sons, Ltd. 

expression, which is regulated by the mating pathway (reviewed in Boeke and Sandmeyer, 1991). On the other hand, the much higher labelling levels of Ty1B ORFs compared to Ty1A might reflect the fact that cDNA was synthesized from the 3′ end of the mRNA. In Ty transposons, all the regular transcripts end just downstream of or inside the B-ORF (Boeke and Sandmeyer, 1991). Therefore, it is possible that more label was incorporated into this part of the sequence, since many of the retrotranscript molecules did not reach the 5′ end of the mRNA. The fact that genomic samples did not show such an effect (Table 2) argues in favour of this hypothesis. However, this would only explain the results for MCY730 but not the ones obtained for T73, in which TyB labelling is not significantly different from TyA. In combination, all these results rather suggest that Ty transcription is in some way defective in T73-producing incomplete molecules. This would result in lower mRNA levels, especially for the 3′-portion of the message (TyB).

The comparison of genomic copy numbers vs. transcript levels (Figure 4) may be a general way to describe a given yeast strain. Especially the information on Ty elements and large gene families, such as the subtelomeric one shown in Figure 4, which are very variable between strains, could be used as a sensitive molecular tool for industrial strain identification, as has been suggested previously (discussed in Pretorius, 2000).

### Acknowledgements

We thank Dr F. Estruch for the gift of strain MCY730 and Dr J. García-Martínez for his help with the analysis of gene families. We acknowledge the excellent sequencing work done by Sistemas Genómicos S.L. (Paterna, Spain) and the technical support by M. Bier. This work was funded by the European Commission as part of the EUROFAN-2 project under contract BIO4-CT97-2294 to J.E.P.-O. and J.D.H.; R.G. was supported by the Spanish programme, Ayudas para la Incorporación a España de Doctores y Tecnólogos, of the Ministerio de Educación y Cultura; J.E.P.-O. was the recipient of a Marie Curie Fellowship of the European Commission.

### References

Adams J, Puskas-Rozca S, Simlar J, Wilke CM. 1992. Adaptation and major chromosomal changes in populations of *Saccharomyces cerevisiae*. *Curr Biol* **22**: 13–19.

Bakalinsky AT, Snow R. 1990. The chromosomal constitution of wine strains of *Saccharomyces cerevisiae*. *Yeast* **6**: 367–382.

Beissbarth T, Fellenberg K, Brors B, *et al.* 2000. Processing and quality control of DNA array hybridization data. *Bioinformatics* **16**: 1014–1022.

Bidenne C, Blondin B, Dequin S, Vezinhet F. 1992. Analysis of the chromosomal DNA polymorphism of wine strains of *Saccharomyces cerevisiae*. *Curr Genet* **22**: 1–7.

Boeke JD, Sandmeyer SB. 1991. Yeast transposable elements. In *The Molecular Biology of the Yeast Saccharomyces*, vol 1, Broach JR, Pringle JR, Jones EW (eds). Cold Spring Harbor Laboratory Press: New York; 193–261.

Brachman CB, Davies A, Cost GJ, Caputo E, Li J, Hieter P, Boeke JD. 1998. Designer deletion strains derived from *Saccharomyces cerevisiae* S288C: a useful set of strains and plasmids for PCR-mediated gene disruption and other applications. *Yeast* **14**: 115–132.

Carlson M, Botstein D. 1983. Organization of the *SUC* gene family in *Saccharomyces cerevisiae*. *Mol Cell Biol* **5**: 2894–2902.

Cavalieri D, Towsend JP, Hartl DL. 2000. Manifold anomalies in gene expression in a vineyard isolate of in *Saccharomyces cerevisiae* revealed bu DNA microarray analysis. *Proc Natl Acad Sci U S A* **97**: 12369–12374.

Codón AC, Benítez T, Korhola M. 1998. Chromosomal polymorphism and adaptation to specific industrial environments of *Saccharomyces* strains. *Appl Microbiol Biotechnol* **11**: 154–163.

Cox KH, Pinchack AB, Cooper TG. 1999. Genome-wide transcriptional analysis in *S. cerevisiae* by mini-array membrane hybridization. *Yeast* **15**: 703–713.

DeRisi JL, Iyer VR, Brown PO. 1997. Exploring the metabolic and genetic control of gene expression on a genomic scale. *Science* **278**: 680–686.

Functional Genome Analysis, DKFZ Heidelberg. URL: **http://www.dkfz-heidelberg.de/funct_genome/index.html**.

Galitski T, Saldanha AJ, Styles CA, Lander ES, Fink GR. 1999. Ploidy regulation of gene expression. *Science* **285**: 251–254.

Goffeau A, Barrell BG, Bussey H, *et al.* 1996. Life with 6000 genes. *Science* **274**: 546–567.

Goto-Yamamoto N, Kitano K, Shiki K, *et al.* 1998. *SSU1-R*, a sulphite resistance gene of wine yeast, is an allele of *SSU1* with a different upstream sequence. *J Ferm Bioengineer* **86**: 427–433.

Guijo S, Mauricio JC, Salmon JM, Ortega JM. 1997. Determination of the relative ploidy in different *Saccharomyces cerevisiae* strains used for fermentation and 'flor' film ageing of dry sherry-type wines. *Yeast* **13**: 101–117.

Hauser NC, Vingron M, Scheideler M, *et al.* 1998. Transcriptional profiling on all open reading frames of *Saccharomyces cerevisiae*. *Yeast* **14**: 1209–1221.

Hoffman CS, Winston F. 1987. A ten-minute DNA preparation from yeast efficiently releases autonomous plasmids for transformation of *Escherichia coli*. *Gene* **57**: 267–272.

Holstege FCP, Jennings EG, Wyrick JJ, *et al.* 1998. Dissecting the regulatory circuitry of a eukaryotic genome. *Cell* **95**: 717–728.

Hughes TR, Marton MJ, Jones AR, *et al.* 2000. Functional discovery via a compendium of expression profiles. *Cell* **102**: 109–126.

Jelinsky SA, Samson LD. 1999. Global response of *Sacchar-*

Copyright © 2001 John Wiley & Sons, Ltd. 

*omyces cerevisiae* to an alkylating agent. *Proc Natl Acad Sci U S A* **96**: 1486–1491.

Jordan IK, McDonald F. 1999. Tempo and mode of Ty evolution in *Saccharomyces cerevisiae*. *Genetics* **151**: 1341–1351.

Kunkee RE, Bisson LF. 1993. Wine-making yeasts. In *The Yeasts: Yeast Technology*, Rose AH, Harrison JS (eds). Academic Press: London; 69–126.

Laboratorio de Chips de DNA de la Universitat de València. URL: **http://scsie.uv.es/chipsdna/.**

Liu H, Styles CA, Fink GR. 1996. *Saccharomyces cerevisiae* S288C has a mutation in *FLO8*, a gene required for filamentous growth. *Genetics* **144**: 967–978.

Liu L, Zeng M, Hausladen A, Heitman J, Stamler JS. 2000. Protection from nitrosative stress by yeast flavohemoglobin. *Proc Natl Acad Sci U S A* **97**: 4672–4676.

Masneuf I, Hansen J, Groth C, Piskur J, Dubourdieu D. 1998. New hybrids between *Saccharomyces sensu stricto* yeast species found among wine and cider production strains. *Appl Environm Microbiol* **64**: 3887–3892.

Naumov GI, Naumova ES, Michels CA. 1995. Genetic variation of the repeated *MAL* loci in natural populations of *Saccharomyces cerevisiae* and *Saccharomyces paradoxus*. *Genetics* **136**: 803–812.

Naumov GI, Naumova ES, Sancho E, Korhola MP. 1996. Polymeric *SUC* genes in natural populations of *Saccharomyces cerevisiae*. *FEMS Lett* **135**: 31–35.

Naumov GI, Turakainen H, Naumova ES, Aho S, Korhola MP. 1990. A new family of polymorphic genes in *Saccharomyces cerevisiae*: α-galactosidase genes *MEL1–MEL7*. *Mol Gen Genet* **224**: 119–128.

Ness F, Aigle M. 1995. *RTM1*: a member of a new family of telomeric repeated genes in yeast. *Genetics* **140**: 945–956.

Park H, Bakalinsky AT. 2000. *SSU1* mediates sulphite efflux in *Saccharomyces cerevisiae*. *Yeast* **16**: 881–888.

Pretorius IS. 2000. Tailoring wine yeast for the new millennium: novel approaches to the ancient art of winemaking. *Yeast* **16**: 675–729.

Puig S, Pérez-Ortín JE. 2000a. Stress response and expression patterns in wine fermentation of yeast genes induced at the diauxic shift. *Yeast* **16**: 139–148.

Puig S, Querol A, Barrio E, Pérez-Ortín JE. 2000b. 'Mitotic recombination as a mechanism of genetic change in yeast during wine fermentations'. *Appl Environ Microbiol* **66**: 2057–2061.

Puig S, Ramón D, Pérez-Ortín JE. 1998. Optimized method to obtain stable food-safe recombinant wine yeast strains. *J Agric Food Chem* **46**: 1689–1693.

Querol A, Huerta T, Barrio E, Ramón D. 1992. Dry yeast strain for use in fermentation of Alicante wines: selection and DNA patterns. *J Food Sci* **57**: 183–185.

Querol A, Ramón D. 1996. The application of molecular techniques in wine microbiology. *Trends Food Sci Technol* **7**: 73–78.

Rachidi N, Barre P, Blondin B. 1999. Multiple Ty-mediated chromosomal translocations lead to karyotype changes in a wine strain of *Saccharomyces cerevisiae*. *Mol Gen Genet* **261**: 841–850.

Sambrook J, Fritsch EF, Maniatis T. 1989. *Molecular Cloning: A Laboratory Manual*. Cold Spring Harbor Laboratory Press: New York.

Sancho ED, Hernández E, Rodríguez-Navarro A. 1986. Presumed sexual isolation in yeast populations during production of sherry-like wine. *Appl Environ Microbiol* **51**: 395–397.

Sherman F, Fink GR, Hicks JB. 1986. *Methods in Yeast Genetics*. Cold Spring Harbor Laboratory Press: New York.

Ter Linde JJM, Liang H, Davis RW, Steensma HY, Van Dijken JP, Pronk JT. 1999. Genome-wide transcriptional analysis of aerobic and anaerobic chemostat cultures of *Saccharomyces cerevisiae*. *J Bacteriol* **181**: 7409–7413.

Wodicka L, Dong H, Mittmann M, Ho M-H, Lockart DJ. 1997. Genome-wide expression monitoring in *Saccharomyces cerevisiae*. *Nature Biotechnol* **15**: 1359–1367.

Woolford JL, Warner JR. 1991. The ribosome and its synthesis. In *The Molecular Biology of the Yeast Saccharomyces*, vol 1, Broach JR, Pringle JR, Jones EW (eds). Cold Spring Harbor Laboratory Press: New York; 587–626.

Zhao X-J, Raitt D, Burke PV, Clewell AS, Kwast KE, Poyton RO. 1996. Function and expression of flavohemoglobin in *Saccharomyces cerevisiae*. *J Biol Chem* **271**: 25131–25138.

Copyright © 2001 John Wiley & Sons, Ltd. 
